# Novel mutations of the *USH2A* gene cause Usher syndrome in five Chinese families

**DOI:** 10.1186/s12886-022-02532-6

**Published:** 2022-07-23

**Authors:** Dongjun Xing, Rongguo Yu, Linni Wang, Liying Hu, Yang Yang, Chang Li, Zhiqing Li, Xiaorong Li

**Affiliations:** Tianjin Key Laboratory of Retinal Functions and Diseases, Tianjin Branch of National Clinical Research Center for Ocular Disease, Eye Institute and School of Optometry, Tianjin Medical University Eye Hospital, 251 Fukang Road, Tianjin, 300384 China

**Keywords:** Usher syndrome, *USH2A*, Targeted exome sequencing, Mutation

## Abstract

**Background:**

Usher syndrome (USH) is a leading disorder of deaf–blindness. The phenotypic and genetic heterogeneity of USH makes the diagnosis of this disorder difficult. However, diagnosis can be facilitated by employing molecular approaches, especially for diseases without pronounced pathognomonic symptoms. Therefore, this study aimed to reveal the genetic defects in five USH patients using clinical targeted exome sequencing (TES).

**Methods:**

USH patients and their family members from five unrelated Chinese USH families were recruited and subjected to TES. Ophthalmic information was obtained for all patients to ensure a meaningful interpretation. The TES data were analysed using an established bioinformatics pipeline to identify causative mutations. Further verification by Sanger sequencing and cosegregation analysis were performed on available family members.

**Results:**

We identified genetic mutations in five USH patients using TES. Seven mutations, four of which were novel, were identified in the *USH2A* gene. One proband (F1-II-3) was found to have a homozygous mutation inherited from nonconsanguineous parents, and another proband (F5-III-1) was found to carry three *USH2A* gene mutations.

**Conclusion:**

In conclusion, the study revealed the importance of TES in the clinical diagnosis of USH patients with variable phenotypes. The correlation between *USH2A* gene mutations and clinical phenotypes will help to refine the clinical diagnosis of USH.

**Supplementary Information:**

The online version contains supplementary material available at 10.1186/s12886-022-02532-6.

Usher syndrome (USH) is an inherited disease that causes deafness and retinitis pigmentosa. It is inherited in an autosomal recessive pattern with both clinical and genetic heterogeneity, and its estimated prevalence is 4–17 per 100,000 people worldwide [1]. USH is the most common cause of combined hearing and vision loss, accounting for more than half of deaf–blind patients [2]. It has been estimated to represent 18% of all retinitis pigmentosa and 5% of all congenital deafness cases. Based on the degree of hearing and vestibular damage, the disease is clinically categorized into three types, each associated with a number of genetic loci. The Usher gene proteins are expressed in the inner retina and ear and perform essential functions in photoreceptor maintenance and sensory hair cell development and function. However, each USH subtype has some clinical variability, with overlapping and atypical presentations described. USH1 type (USH1) is the most severe subtype, characterized by severe-to-profound bilateral congenital sensorineural hearing loss, prepubertal retinitis pigmentosa (RP) and vestibular areflexia, accounting for approximately 25–44% of all USH cases [3]. USH2 type (USH2) is the most common subtype of the disease. Patients present with congenital moderate-to-severe sensorineural deafness, normal vestibular function, and retinitis pigmentosa beginning at 10–20 years of age. USH3 type (USH3) is a rare form characterized by postlingual deafness, teenage-onset RP and varying degrees of vestibular dysfunction. It accounts for approximately 2–4% of all USH cases, although it is particularly prevalent among the Ashkenazi Jewish and Finnish populations [4]. Approximately 70% of USH2 cases are caused by mutations in the *USH2A* gene, and more than 400 such mutations have been reported to date. Here, clinical examination was performed on 5 families with Type 2 USH, and gene mutation detection was carried out through target region capture sequencing, and all of the families were found to carry mutations in the *USH2A* gene. The results are now reported as follows.

## Methods

### Ethics statement

The study adhered to the tenets of the Declaration of Helsinki and was approved by the Ethics Committee of Tianjin Medical University Eye Hospital (2018KY-13). Written informed consent was received from all parents and their family members after detailed explanation of the genetic study was provided. We collected the clinical and familial pedigree from each proband.

### Study samples and clinical assessment

Five unrelated individuals and their unaffected family members from 5 USH2 families were admitted to Tianjin Medical University Eye Hospital, Tianjin, China. Comprehensive ophthalmic examinations, including routine eye tests, best corrected visual acuity (BCVA), ultra-widefield fundus photography (UW-FP), fundus autofluorescence (FAF), spectral-domain optical coherence tomography (OCT), perimetry and electroretinography (ERG), were carried out in the patients. All patients showed typical RP fundus manifestations accompanied by hearing impairment and normal vestibular function.Pedigrees were drawn after collection of a detailed family history and consanguinity information through personal interviews with family members.

### DNA library preparation and targeted gene capture and sequencing

Peripheral vein blood (3–5 ml) was collected from all study subjects using an EDTA Vacutainer. Genomic DNA was extracted from the leukocytes using a DNA Extraction Kit (TIANGEN, Beijing, China). We used 3 µg genomic DNA for the indexed DNA libraries. DNA fragments of 350 bp to 450 bp, including adapter sequences, were selected for the DNA libraries. Targeted exome sequencing (TES) was performed for 5 study participants. A total of 381 known genes associated with inherited retinal diseases (IRDs), including USH, were selected by a gene capture strategy using the GenCap custom enrichment kit following the manufacturer’s protocol [5]. The biotinylated capture probes (80–120-mer) were designed to tile all of the exons with nonrepeated regions. Using the Illumina HiSeq 2000 platform, the enriched libraries were sequenced as paired-end reads of 100 bp.

## Bioinformatic analysis

Sequencing data were postprocessed using Bcl2Fastq software for base calling and raw data generation. Low-quality reads were filtered out using a quality score ≥ 20. Short Oligonucleotide Analysis Package (SOAP) aligner software was then used to align the clean reads to the reference human genome (HG19). Subsequently, single nucleotide polymorphisms (SNPs) were determined using the SOAP SNPs program, and deletions and insertions (InDels) were detected using Genome Analysis Toolkit software. We annotated the identified SNPs and InDels using the Exome-assistant program (http://122.228.158.106/exomeassistant) and used MagicViewer to view the short read alignment to confirm the candidate SNPs and InDels. The pathogenicity of non-synonymous variants was predicted using four in silico tools: PolyPhen (http://genetics.bwh.harvard.edu/pph2/), Sorting Intolerant From Tolerant (SIFT, http://sift.jcvi.org/), Protein Analysis Through Evolutionary Relationships (PANTHER, www.pantherdb.org) and Pathogenic Mutation Prediction (PMut, http://mmb.pcb.ub.es/PMut/).

### Expanded familial validation and protein function prediction

Whole-blood genomic DNA from the five probands was subjected to TES and analysis. Short and low-quality reads were removed from the sequencing data and then compared with HG19 to extract SNPs, Indels and missense mutations. SIFT, Polyphen and Mutation Taster were used to predict the effects of missense amino acid mutations on protein function, and GERP +  + was used to predict the conserved amino acid mutation sites. The data were also compared to the sequences collected in the Human Genome Mutation Database, the 1000 Genomes Project, ExAC, ClinVar, and ESP6500si. Suspected pathogenic mutations were verified by Sanger sequencing and intrafamily cosegregation analysis. The coding exons containing the candidate mutations were amplified using Ex Taq DNA polymerase (Takara, Dalian). The PCR samples were sequenced on an ABI 3500 Genetic Analyzer (Applied Biosystems). Data were analysed and interpreted according to the American College of Medical Genetics and Genomics guidelines.

## Results

### Phenotypic determination

**Fig. 1:**
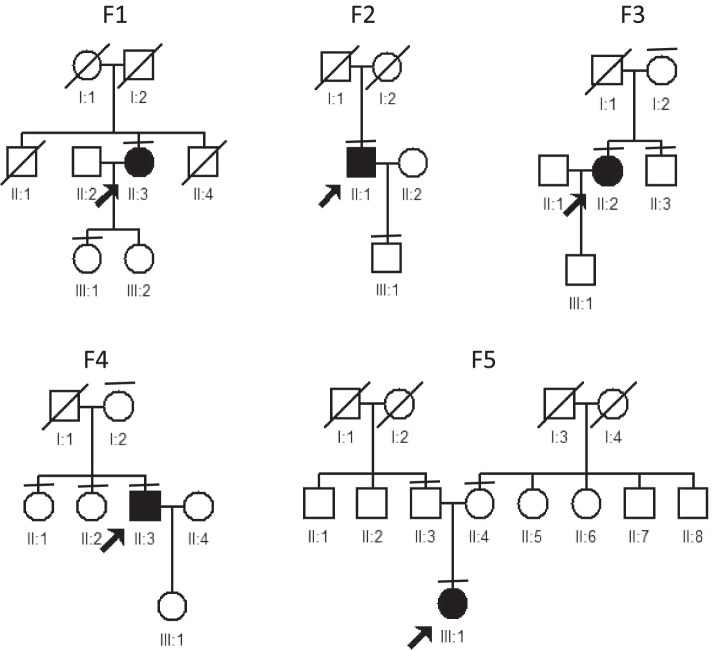
Pedigrees of Usher syndrome families. Closed black symbols represent patients, and open symbols indicate unaffected subjects. A slash indicates a deceased person. Arrows indicate the probands. Bars over symbols indicate examined subjects

**Fig. 2:**
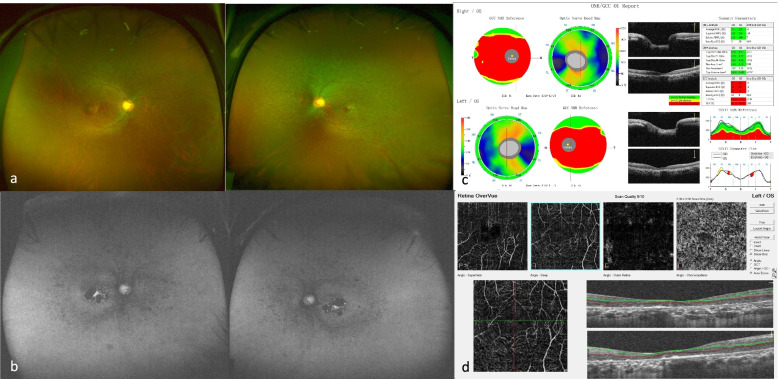
Clinical phenotype of F3-II-2. UW-FP demonstrated midperipheral pigment migration and attenuated retinal vessels (**a**). FAF demonstrated midperipheral and macular patchy hypoautofluorescence (**b**). The macular GCC was thinned, while the RNFL was normal in both eyes (**c**). Macular OCTA demonstrated a decreasing vascular flow density (superficial and deep). The FAZ of both the superficial plexus and deep capillary plexus was enlarged, and central foveal thinning and loss of photoreceptors were evident (**d**)

**Fig. 3:**
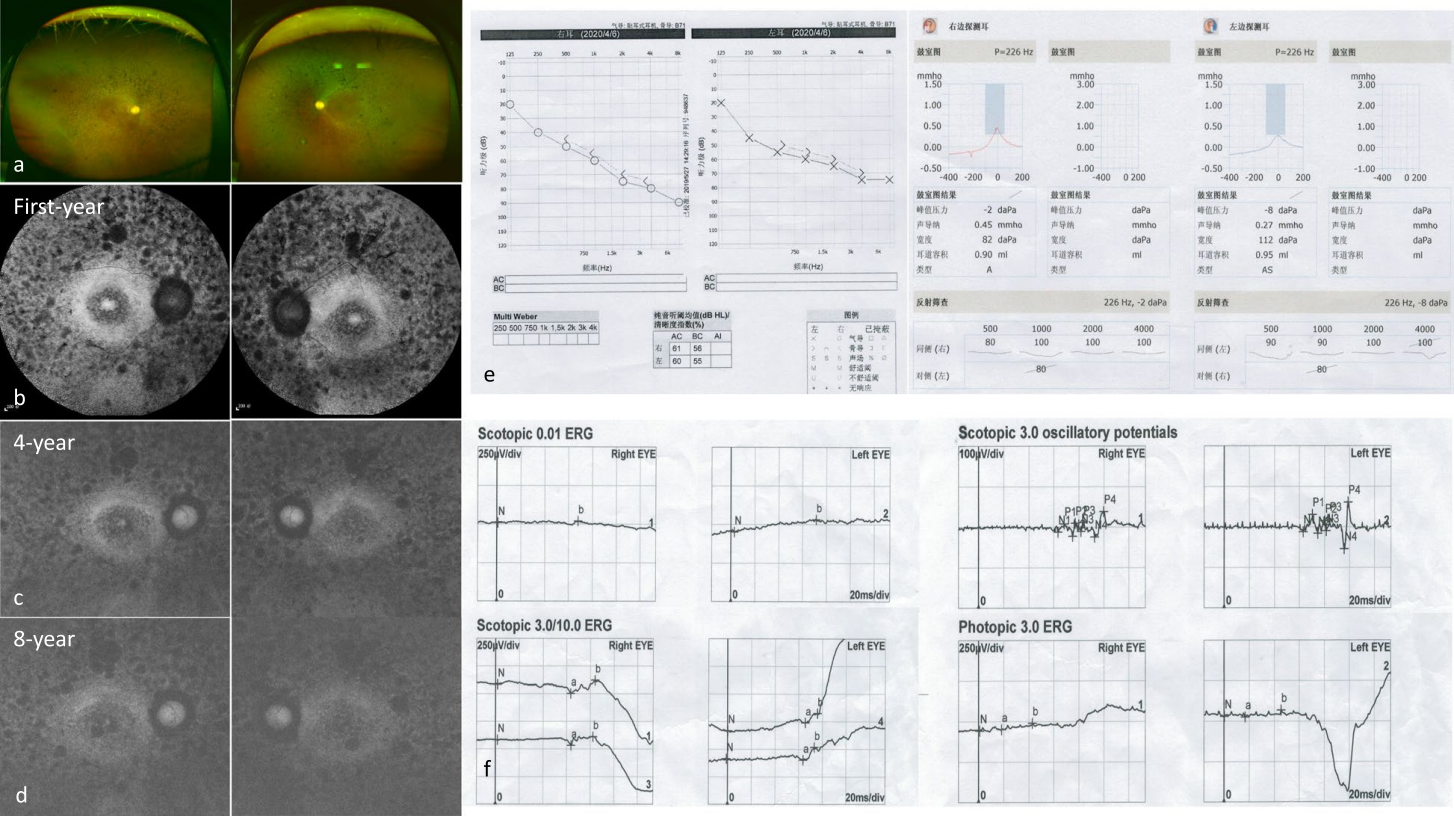
Clinical examination of F2-II-1 and F5-III-1. Fundus photos of F2-II-1 indicate the atrophic lesion around the retina (**a**). FAF images of F2-II-1 show that the parafoveal autofluorescence ring faded away in eight years (**b**, **c**, **d**). Images showed that F5-III-1 was able to achieve good hearing with hearing aids (**e**). Scotopic and photopic ERG testing showed an extinguished rod response in the F5 proband (**f**)

USH followed an autosomal recessive inheritance pattern in all patients (Fig. 1). Bone-spicule hyperpigmentation, attenuated arteries and sensorineural deafness signs (Figs. 2a and 3a) were consistently observed in all patients. Based on the classification of visual acuity according to the World Health Organization (International Classification of Disease 11, 2018), three patients were legally blind, and one patient had severe hearing impairment. Optical coherence tomography (OCT) imaging suggested significantly diminished macular ganglion cell complex (GCC) thickness, reduced macular thickness and disorganized outer segment structure, while the RNFL was normal in both eyes (Fig. 2c). Optical coherence tomography angiography (OCTA) revealed a decreasing macular capillary flow density in the superficial and deep capillary plexus, while the foveal avascular zone (FAZ) was also enlarged (Fig. 2d). Fundus autofluorescence (FAF) images collected from patients are shown and demonstrated midperipheral and macular patchy hypoautofluorescence (Fig. 2b). FAF images of F2-II-1 show that the parafoveal autofluorescence ring faded away in eight years (Fig. 3b, c and d). ERG showed no response from the rod cells (Fig. 3f). One patient (F5-III-1) had a hearing aid when she was five years old (Fig. 3e). The clinical findings of each patient are summarized in Table 1.

**Table 1:**
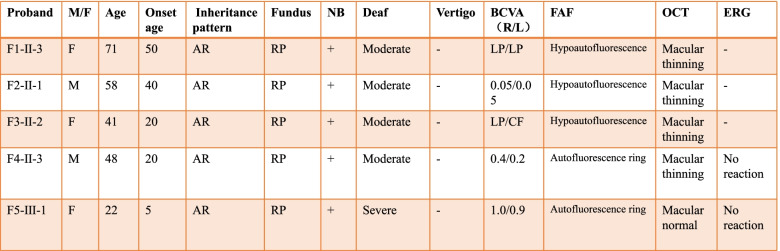
Clinical manifestations of the probands

### Targeted exome sequencing data analysis

Targeted exome sequencing (TES) of 381 genes implicated in inherited retinal degeneration (IRDs) was performed. On average, 2.7 GB of raw data were generated per targeted exome, of which 90.30% to 94.58% had quality scores above Q30. Each targeted exome contained approximately 5,554,000 reads, while an average of 4,502,000 reads remained after adapter trimming. Overall, the mapped reads achieved more than 99.89% coverage of the targeted regions. The average sequencing depths of the targeted regions ranged from 274.42X to 754.24X (Supplemental Table S1). Overall, 99.45% to 99.77% of targeted exons exhibited coverage > 10X and 97.63% to 99.49% had coverage > 20X. Approximately 3,000 variants were identified for each sample by using the SOAPsnp program [6]. After excluding the SNPs released of the 1000 Genomes Project with a MAF > 0.05 and variants reported in HapMap 28, the variants were further narrowed down to 26–223, respectively (Supplemental Table S2, S3, S4, S5 and S6). USH is inherited in an autosomal recessive pattern, so homozygous and compound heterozygous variants were considered for analysis. Among the 370 substitutions, fewer than 20 candidate mutations were further selected by using functional prediction and conservation analysis and consistency with the genetic transmission mode. By following the described sample filtering strategy, we successfully identified candidate mutations in all patients.

### Confirmation of pathogenic mutations by familial validation and Sanger sequencing

**Fig. 4:**
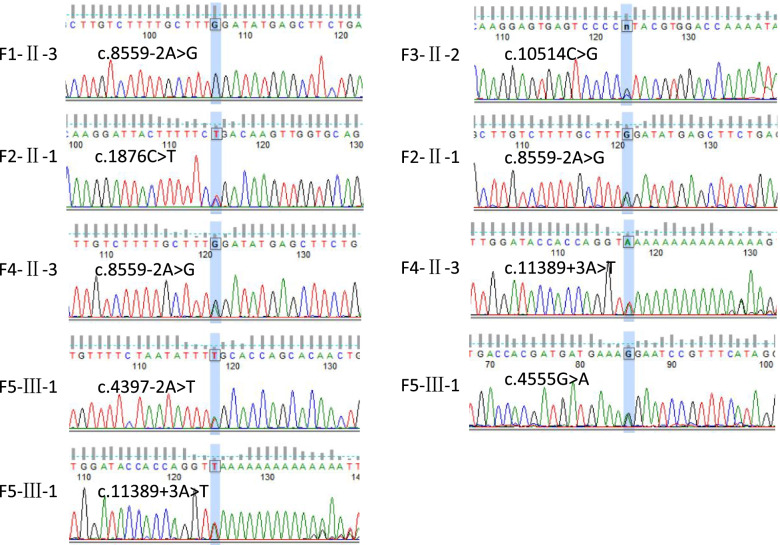
The Sanger sequencing results of all patients

We identified disease-causing mutations in five families with sporadic USH2 (Table 2). In the five pedigrees, the probands were all diagnosed with typical USH2 based on their clinical features. In family F1, the proband (F1-II-3) carried a homozygous *USH2A* mutation (c.8559-2A > G, splice site), which was subsequently transmitted to the healthy (heterozygous) daughter (F1-III-1), and we reconfirmed that no consanguineous marriage had occurred. In family F2, the TES results from the proband showed two compound heterozygous *USH2A* mutations (p.R626X and c.8559-2A > G), and the unaffected son harboured a heterozygous *USH2A* mutation (p.R626X). In pedigree F3, the proband (F3-II-2) was found to carry two mutations (p.P3505R and exon 61dup). This condition was further confirmed by intrafamilial validation with Sanger sequencing and MLPA. One mutation (p.P3505R) was transmitted from the unaffected mother (F3-I-2), and it is conceivable that the mutation (*USH2A* exon 61dup) was inherited from her (now-deceased) father (F3-I-1), as her unaffected brother also harboured this mutation. In family F4, a compound heterozygous mutation (c.8559-2A > G and c.11389 + 3A > T) was identified in the proband (F4-II-3). Sanger sequencing of the coding region further confirmed that the mutation c.8559-2A > G was derived from an unaffected mother (F4-I-2), while two unaffected siblings (F4-II-1 and F4-II-2) had no mutation. In the last pedigree, F5, the proband (F5-III-1) was found to carry three *USH2A* gene mutations. Two novel heterozygous mutations, c.4397-2A > T and c.4555G > A (p.G1519R), were confirmed in her unaffected father (F5-II-3), and the same two mutations (c.4397-2A > T and c.4555G > A) were present in the maternal allele. Another splicing mutation, c.11389 + 3A > T, was identified in the unaffected mother (F5-II-4). Thus, all identified mutations were successfully validated in five USH families via expanded TES, Sanger sequencing (Fig. 4) and cosegregation analysis.

**Table 2:**
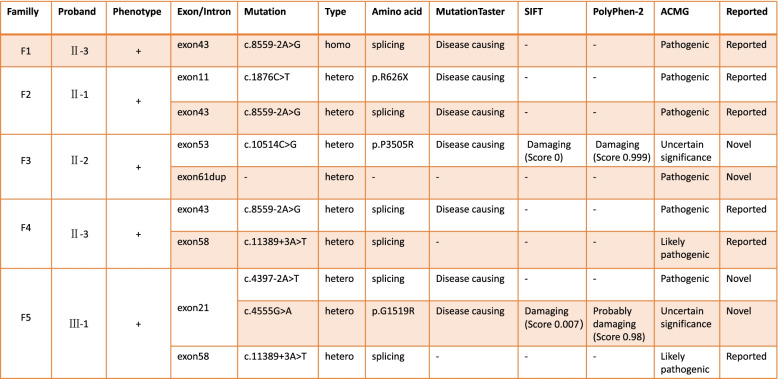
Identified mutations in the *USH2A* gene

## Discussion

Usher syndrome is a severe genetic disorder that causes combined hearing and vision loss [7]. USH was first described by Scottish ophthalmologist Charles Usher, and its prevalence worldwide ranges from 1 to 4 per 25,000 people [1, 8]. Based on its diverse clinical symptoms, the disease can be classified into three types. USH type 1 (USH1) is defined by RP onset within the first decade of life, congenital severe-to-profound deafness and vestibular areflexia. USH type 2 (USH2) is defined by RP onset within the second decade of life, congenital moderate-to-severe hearing loss and normal vestibular function. USH type 3 (USH3) is characterized by progressive postlingual deafness, sporadic vestibular dysfunction and variable onset of RP. Fifteen USH genes have been identified, and three loci have been mapped in RetNet (https://sph.uth.edu/retnet/sum-dis.htm). Considering that these genes contain a large number of coding exons, TES represents a rapid, high-throughput and efficient screening strategy for RP and USH [9]. We used TES to target 381 known causative genes of inherited retinal disorders as an efficient way to perform gene screening [5].

*USH2A* gene mutations represent the most frequent cause of Usher syndrome and account for 57–79% of USH2 cases. Simultaneously, *USH2A* gene mutations cause 12–25% of autosomal recessive nonsyndromic RP (nsRP) and are its leading genetic cause [10–12]. The *USH2A* gene encodes multiple usherin isoforms due to alternative splicing, including short isoform A and very large isoform B. The long isoform B is predominant in the retina and cochlea. Isoform B contains an intracellular region, a short transmembrane domain and a long extracellular domain with 72 exons, leading to a 5202-aa matrix protein [13]. In this study, *USH2A* gene mutations predisposed patients to the disease in five USH families, suggesting a high frequency of *USH2A* mutations in Chinese USH patients. Meanwhile, the parents of the five probands all came from unaffected families and were not consanguineous. In the five USH families, *USH2A* gene mutations predisposed the patient to the disease, suggesting a high prevalence of *USH2A* in the USH population of China. In family F1, the proband (F1-II-3) from a nonconsanguineous family was eventually found to carry a homozygous mutation (c.8559-2A > G). In family F5, the young patients (F5-III-1) were identified to carry three mutations in the *USH2A* gene, which may have contributed to the premature damage of hearing compared to other patients. It is worth emphasizing that four mutations found in this study have not been reported previously (Table 2). Our study indicates a distinctive USH-related mutation spectrum in the Chinese population and may further require much more cohort research.

In the present study, we successfully identified mutations in five Chinese USH2 families by TES. TES could serve as a rapid and efficient screening strategy. Combining TES and clinical information can help to obtain more accurate diagnoses for USH patients. We reported four novel *USH2A* gene mutations, broadening the mutation spectrum of USH2 and providing a new locus for gene therapy.

## Supplementary Information

**Additional file 1.**

**Additional file 2.**

**Additional file 3.**

**Additional file 4.**

**Additional file 5.**

**Additional file 6.**

## Data Availability

All data are fully available without restriction, and please contact corresponding Author the if someone wants to request the data from this study. The TES data of all patients in additional supplementary files: Table S2, Table S3, Table S4, Table S5, Table S6.

## Abbreviations

USH: Usher syndrome
TES: Targeted exome sequencing
RP: Retinitis pigmentosa
OCT: Optical coherence tomography
OCTA: Optical coherence tomography angiography
IRD: Inherited retinal disease
BCVA: Best-corrected visual acuity
UW-FP: Ultra-widefield fundus photography
FAF: Fundus autofluorescence
ERG: Electroretinography
SOAP: Short Oligonucleotide Analysis Package
SNPs: Single nucleotide polymorphisms
PCR: Polymerase chain reaction
GCC: Ganglion cell complex
FAZ: Foveal avascular zone
nsRP: Nonsyndromic RP
