# Supplementary material for: Novel mutations of the USH2A gene cause Usher syndrome in five Chinese families
Source: BMC Ophthalmol. 2022 Jul 23;22:317. doi: 10.1186/s12886-022-02532-6 (PMC9308926; doi:10.1186/s12886-022-02532-6)
Supplement: Supplementary file 1 — Additional file 1. [file 12886_2022_2532_MOESM1_ESM.doc]

**Table S1. Quality of the targeted exome sequencing of the probands.**

| **Sample** | **Aligned (%)** | **Fraction of effective bases on target** | **Average sequencing depth on target** | **Fraction of target covered with at least 4X** | **Fraction of target covered with at least 10X** | **Fraction of target covered with at least 20X** |
| --- | --- | --- | --- | --- | --- | --- |
| F1-II-3 | 99.88% | 19.89% | 274.42 | 99.74% | 99.47% | 98.97% |
| F2-II-1 | 99.89% | 18.57% | 426.4 | 99.81% | 99.67% | 99.38% |
| F3-II-2 | 99.90% | 65.01% | 702.35 | 99.93% | 99.77% | 99.44% |
| F4-II-3 | 99.94% | 56.66% | 386.28 | 98.96% | 98.45% | 97.63% |
| F5-III-1 | 99.98% | 29.14% | 754.24 | 99.87% | 99.70% | 99.49% |
